# Is Camphor the Future in Supporting Therapy for Skin Infections?

**DOI:** 10.3390/ph17060715

**Published:** 2024-05-31

**Authors:** Anna Duda-Madej, Szymon Viscardi, Małgorzata Grabarczyk, Ewa Topola, Joanna Kozłowska, Wanda Mączka, Katarzyna Wińska

**Affiliations:** 1 Department of Microbiology, Faculty of Medicine, Wroclaw Medical University, Chałubińskiego 4, 50-368 Wrocław, Poland; 2 Faculty of Medicine, Wroclaw Medical University, Ludwika Pasteura 1, 50-367 Wrocław, Poland; szymon.viscardi@student.umw.edu.pl (S.V.); ewa.topola@student.umw.edu.pl (E.T.); 3 Department of Food Chemistry and Biocatalysis, Faculty of Biotechnology and Food Science, Wrocław University of Environmental and Life Sciences, C.K. Norwida 25, 50-375 Wrocław, Poland; malgorzata.grabarczyk@upwr.edu.pl (M.G.); joanna.kozlowska@upwr.edu.pl (J.K.); katarzyna.winska@upwr.edu.pl (K.W.)

**Keywords:** camphor, antibacterial activity, antifungal activity, drug resistance, skin infections, wound healing, monoterpenoids, natural compounds

## Abstract

The aim of this review is to present the potential application of camphor—a bicyclic monoterpene ketone—in the prevention of skin infections. Skin diseases represent a heterogeneous group of disorders characterized by prolonged symptoms that significantly diminish the quality of life. They affect the dermis, the epidermis, and even subcutaneous tissue. They very often have a bacterial or fungal background. Therapy for dermatological skin disorders is difficult and long-term. Therefore, it is important to find a compound, preferably of natural origin, that (i) prevents the initiation of this infection and (ii) supports the skin’s repair process. Based on its documented anti-inflammatory, antibacterial, antifungal, anti-acne, anesthetic, strengthening, and warming properties, camphor can be used as a preventative measure in dermatological infectious diseases and as a component in medical and cosmetic products. This work discusses the structure and physicochemical properties of camphor, its occurrence, and methods of obtaining it from natural sources as well as through chemical synthesis. The use of camphor in industrial preparations is also presented. Additionally, after a detailed review of the literature, the metabolism of camphor, its interactions with other medicinal substances, and its antimicrobial properties against bacteria and fungi involved in skin diseases are discussed with regard to their resistance.

## 1. Introduction

Today, a growing interest in the use of natural compounds in various therapies or medical treatments can be observed. Camphor, an organic compound that belongs to the terpene group, is a great example. It is obtained from the wood of the camphor cinnamon tree that grows in Japan, China, Vietnam, Asia, Africa, Sri Lanka, Australia, Canada, and the United States. In its solid form, it is a white powder with a very intense odor that easily converts to a gas. The most commonly used form is camphor oil (*Oleum camphoratum*).

Camphor is not only a fragrant compound, as due to its properties it has also found use in the therapy of skin diseases of bacterial and fungal etiology. Camphor oil is used to support skin problems such as acne, eczema, inflammation, ulcers and wounds, psoriasis, and fungal foot infections [1,2,3,4,5]. Camphor provides support for this type of infection as it has been shown to have a local anesthetic effect [6]. Furthermore, it is effective against swelling and has a unique ability to remove keratinized epidermis [7,8]. As a result, it speeds up wound-healing time and facilitates faster regeneration of the damaged area. However, it is essential to note that pure camphor, in a dose of 2 g, causes very severe poisoning. Additionally, the amount of 4 g is considered a lethal dose (calculated as >30 mg/kg of body weight in oral use) [9,10]. It should also not be applied directly to open wounds as it can easily penetrate the bloodstream and cause toxic effects on the body. Therefore, from a medical point of view, using camphor in the form of oil, preferably highly diluted, is much safer.

Camphor has also found use in cosmetology as an ingredient in ointments, lotions, and depilatories. In addition to pharmaceuticals, it is used to make gels to prevent insect bites or deliver analgesic, anti-itch, and skin cooling effects. It is also used for embalming corpses [11].

The skin is a multifunctional organ that protects tissues from the effects of environmental factors. It serves as a barrier against minor trauma that causes violation/destruction of its integrity and exposes its deeper layers to infection. crucial factors in this process are the malfunctioning of the immune system (reduced cellular and humoral immunity), coexisting diseases (e.g., diabetes),and the pathogenic properties of bacteria involved in inflammation processes [12]. In the case of a properly functioning immune response, keratinocytes prevent bacteria from taking up residence for a long time, promoting only transient colonization. In contrast, under conditions of immune imbalance, inflammation develops. Initially, it involves only the surface layer of the epidermis. However, under favorable conditions, it can also occupy the deeper layers of the skin and even progress to a generalized infection, increasing the risk of the patient’s death. Skin infections can be caused by bacteria (i.e., *Staphylococcus* spp., *Streptococcus* spp., *Corynebacterium* spp., *Spirochaeta* spp., *Actinomyces* spp., and *Mycobacterium* spp.), as well as by fungi (mainly *Candida* spp. and dermatophytes) [13,14,15,16,17,18,19]. Nevertheless, the most commonly observed infections are staphylococcal (e.g., periungual inflammation, figwort, and boils), streptococcal (e.g., roseola) and mixed staphylococcal/streptococcal (e.g., impetigo) [20]. 

Skin disorders can occur either as primary infections or as secondary subinfections of pre-existing dermatosis. In primary infections, the inflammatory process is localized in the dermis (e.g., erysipelas, impetigo), in the hair follicle (folliculitis, furuncle), in the area around the nail (paronychia), or in the subcutaneous tissue (cellulitis) [21]. On the other hand, secondary infections include wound contamination due to injuries, bites, burns, pressure sores, and surgical procedures [22]. 

Microorganisms, both those that are constantly present and those transiently colonizing the skin and mucous membranes, are a serious source of hospitalized infections. The problem becomes even more alarming when multi-drug resistant microorganisms are involved. The presence of *Staphylococcus aureus*, particularly methicillin-resistant (MRSA), significantly increases the risk of surgical site infections, as this species is a key etiological agent of hospital-acquired infections [23]. Research by Bekka-Hadji et al. confirms the effectiveness of camphor against MRSA strains [24]. Moreover, microorganisms that naturally reside on and colonize the skin, including those that cause dermatological infections, are commonly implicated in catheter-related infections. As a results of their ability to adhere to artificial surfaces, they form a unique microenvironment called a biofilm. This directly paves the way for bloodstream infections (BSIs), which ultimately lead to sepsis [25]. This situation is particularly dangerous in the case of secondary infections (i.e., burn wounds, post-operative wounds), whose etiological agents are hospital-acquired MDR strains, mainly those of the ESKAPE group, that is, *Enterococcus faecium*, *Staphylococcus aureus*, *Klebsiella pneumoniae*, *Acinetobacter baumannii*, *Pseudomonas aeruginosa*, and *Enterobacter* spp. [26]. These pathogens represent a new paradigm related to pathogenesis, transmission, and resistance, and they are particularly dangerous from the point of view of the effectiveness of therapy for nosocomial infections. The fight against these microorganisms, and consequently the need to eradicate the biofilms formed by them, is even crucial to the effectiveness of therapy. Camphor’s antimicrobial capabilities appear to be one of the available solutions [27]. A recent study is very promising and demonstrates the synergistic effects of camphor with antibiotics. It is proven that this compound not only contributes to lowering the MIC (minimal inhibitory concentration) values of antimicrobial drugs (i.e., penicillins, cephalosporins, carbapenems, monobactams, aminoglycosides, and quinolones), but also returns antibiotic sensitivity to strains that previously showed resistance to them [28,29].

One of the most dangerous contributors to bacterial superinfection is atopic dermatitis (AD). Skin infections are listed as an integral part of this condition. In people suffering from AD, the integrity of the stratum corneum is damaged and, in addition, the alkaline reaction of the skin surface results in a decrease in its antimicrobial activity. Furthermore, in these individuals a decrease in lipid content is observed in the epidermis, which hinders water binding, leading to faster moisture loss and crack formation. The skin becomes vulnerable and susceptible to microorganisms in the environment. This is particularly dangerous among children and adolescents, as recent studies conducted in 14 countries have shown an increasing trend of AD among those aged 6–14 years [30]. These data are in accordance with those presented in the Global Report on Atopic Dermatitis, which reports that AD affects up to 20% of children, 10% of young adults (20–25 years old) and 1–3% of adults [31]. The situation becomes particularly dangerous when it is additionally associated with the hospital environment, which favors the spread of multi-drug resistant strains. Retrospective studies conducted by Grossi et al. in hospitals in six European countries provide direct evidence that acute bacterial skin and skin structure infections (ABSSSI), due to their high treatment costs, constitute a significant burden on healthcare [32]. Therefore, the prevention of skin infections, including AD, is crucial not only during exacerbation, but also primarily during remission. The introduction of preparations containing compounds of natural origin, especially camphor, would: (i) strengthen the skin barrier, and accelerate its reconstruction; and also (ii) replenish lipid deficiencies and make the skin more resistant to microbial attacks. In addition, camphor exhibits soothing properties for irritations and prevents inflammatory conditions. Studies by Kang et al. have given the green light to treating this condition using leaves from *Cinnamomum camphora*. These researchers showed that the leaves have a calming effect on the production of inflammatory chemokines in vitro, resulting in relief of symptoms of dermatitis in vivo [4]. Combining the positive effects of camphor’s antimicrobial properties with its therapeutic properties would be key in improving the quality of life for patients suffering from AD while simultaneously preventing potential skin superinfections of bacterial and/or fungal etiology.

In our review, we have focused on the positive impact of camphor in preventing the formation of difficult-to-heal wounds infected with strains exhibiting high resistance to antibiotics. Addressing the issues of hospital-acquired infections, we primarily focused on strains that reside in the hospital environment and exhibit the ability to acquire resistance easily while also forming biofilms. These microorganisms pose a real threat in hospitals around the world. 

## 2. Camphor’s Chemical Properties and Methods of Production

**Figure 1:**
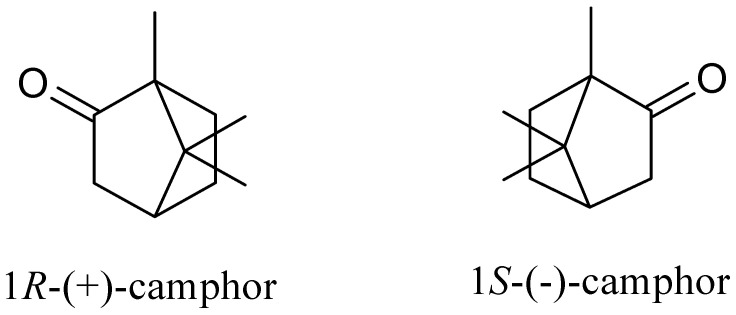
Structure of camphor isomers.

Camphor is a white solid with a characteristic odor and pungent taste, which sublimates at room temperature. It does not dissolve in water, although it dissolves well in organic solvents such as diethyl ether, ethanol, and chloroform. Chemically, camphor (1,7,7-trimethylbicyclo[2.2.1]heptan-2-one) is a cyclic terpenoid ketone with the general formula C_10_H_16_O that occurs as two isomers: R-(+)-camphor and S-(-)-camphor (Figure 1) [33,34].

Camphor can be obtained by chemical synthesis both as a racemic mixture and as a pure S-(-) isomer. The most common substrate used during synthesis is α-pinene. This compound is isomerized to camphene through the Wagner–Meervein rearrangement, which is a nucleophilic rearrangement involving 1,2-migration of a ring carbon atom in a bicyclic molecule. In the next step, camphene is reacted with formic or acetic acid, resulting in an isobornyl ester. This ester undergoes hydrolysis to isoborneol, which upon oxidation is converted to (±)-camphor [35].

An alternative method is to use dihydrocarvone for synthesis. Dihydrocarvone is reacted with isopropenyl acetate in the presence of p-toluenesulfonic acid as a catalyst. The ester formed in this reaction closes to (±)-camphor when subjected to the action of boron trifluoride [36].

Obtaining S-(-)-camphor requires a combination of classical chemical synthesis methods and the use of enzymes. The α-pinene chemical was used as a substrate, from which an isobornyl ester was obtained. This ester was then subjected to kinetic separation using an esterase derived from *Burkholderia gladioli* to obtain pure (+)-isoborneol, from which, after oxidation, (-)-camphor was obtained [37].

Another method is to use Oxone, which is a mixture of the three salts 2KHSO_5_, 3KHSO_4_, and 3K_2_SO_4_, in which the active agent is potassium peroxymonosulfate, KHSO_5_, as the oxidizing agent. In this case, (1*S*)-borneol is used for the reaction, which, after oxidation with Oxone, is converted to (-)-camphor [38].

## 3. Camphor Occurrence

Naturally occurring camphor is extracted from the wood of the camphor laurel (*Cinnamomum camphora* L.), which is native to eastern and southern Asia and now grows worldwide [34]; from the camphor tree *Dryobalanops aromatica*, which grows in Sumatra [39]; as well as from the camphor tree found in Africa *Ocotea usambarensis* [40]. As noted previously, camphor is insoluble in water; in addition, it is characterized by a high boiling point of 204 °C. These properties are used in the isolation of this monoterpenoid, because it is obtained by steam distillation followed by sublimation.

The essential oil (EO) extracted from camphor tree leaves is a mixture of volatile compounds, mainly terpenes, and is widely used in pharmaceutical, industrial, and environmental applications. Camphor tree EO extraction methods mainly include (i) steam extraction, (ii) organic solvent extraction, (iii) ionic liquid combined with microwave-assisted extraction, (iv) microwave extraction without solvents, (v) cold-pressed extraction, (vi) subcritical CO_2_ extraction, (vii) ultrasound-assisted extraction, and (viii) microwave-assisted extraction, as well as the (ix) supercritical fluid extraction method. The latter method is used to split camphor tree leaves into a soluble fraction SC-CO_2_ [41,42].

It is important to note that the amount of camphor in plants varies depending on the species and part of the plant. Studies have shown that *C. camphora* leaf oil is composed mainly of camphor, which accounts for as much as 93.1%, whereas in wood oil camphor is found alongside 1,8-cineol (19.8%), α-terpineol (6.2%), and safrole (3.2%), and it accounts for only 53.2% of the wood oil content [43].

Also, depending on the particular chemotype, EO extracted from the same plant species, i.e., from *C. camphora*, can contain from as much as 74% camphor (camphor chemotype) [44] down to only 5% (linalool chemotype) [45]. On the other hand, the cineole chemotype does not contain this compound [33].

Table 1 shows the camphor contents of essential oils and extracts from various plants.

## 4. Camphor as an Additional Ingredient in Products

Camphor is used as an ingredient in various ointments used as a broad-spectrum remedies. As pure camphor can be dangerous in overdose, in order to take advantage of its health-promoting properties it can only be used as one of the ingredients of appropriate medications.

One such ointment is Tiger Balm^®^, which is used as a herbal analgesic for external application. Two variants of Tiger Balm^®^ are known, namely Red TB^®^ and White TB^®^. The Red variant is recommended for use for muscle pain, whereas the White variant is recommended for use for tension headaches. Both variants contain 11% camphor in their composition. In addition, the main ingredients in both formulations for Red TB^®^ and White TB^®^ are, respectively, menthol (10.0% and 8.0%), clove oil (5.0% and 1.5%) and cajuput oil (7.0% and 13.0%) [65]. Another painkilling ointment is Bengay, which contains camphor, methyl salicylate, and menthol [66]. In addition, the main active ingredients of Vicks VapoRub^®^ ointment are camphor (5%), turpentine oil (5%), L-menthol (2.75%), and eucalyptus oil (1.5%), thymol and cedarwood oil.

This ointment is used as an adjunct in the treatment of colds and other upper respiratory tract diseases in the form of rubbing the chest and back [67]. The feeling of better breathing after using this ointment is mainly due to the action of camphor and menthol, which interact with the cold receptors in the nose, causing a feeling of cold by constricting the nasal mucosa, which in turn facilitates breathing. A small amount of Vicks VapoRub^®^ can also be dissolved in hot water and used for inhalation [67]. In 2023, Stinson et al. conducted research using rhinovirus serotype-16 (RV16)-infected A549 airway epithelial cells, in which they showed that Vicks VapoRub^®^ modulates specific TRP (transient receptor potential) receptors and reduces the ATP release induced by RV16 [67].

There are reports in the scientific literature indicating the possibility of using Vicks VapoRub^®^ also in the treatment of nail fungus. During in vitro tests, the ingredients of the ointment showed a broad spectrum of activity and were active against, among other microbes, *Candida*, *Aspergillus*, and some dermatophytes species, which encouraged further research. However, there were some errors in a 48-week clinical trial conducted by the Family Medicine Group in 2011. Unlike the phase III clinical trials for toenail fungus, this study did not exclusively enroll patients with cultured dermatophytes such as *Trichophyton rubrum* or *Trichophyton mentagrophytes* and did not limit the percentage of affected toenails to 50 or 60% [68]. At baseline, 10 of the 18 patients had more than 60% nail involvement, and some had 89% or even 100% involvement [69]. In the case of completely dystrophic nails, a 48-week treatment period may not be sufficient. However, adding a method such as nail cleaning could demonstrate the synergistic effect of this procedure and the ointment used. Additionally, in this study some patients used the ointment daily, although others used it only three to five times a week. Therefore, it cannot be ruled out that the ointment caused the formation of a more moisturized nail unit, which gives the appearance of a healthier nail, instead of actually curing the fungus [68].

In 2016, a study was conducted on the effectiveness of Vicks VapoRub^®^ ointment in the treatment of nail fungus in people infected with HIV. In the case of these patients, it is very difficult to select an effective treatment method due to possible interactions of the therapy used with antiretroviral drugs. Of the 20 study participants, 94% of patients reported satisfaction with their toenail health and their overall treatment results at the 24-week follow-up visit [70].

Camphor has also found use as an ingredient in various types of soaps. As early as the 19th century, the so-called “medicinal soap” was used, which included camphor, and was used for the therapy of frostbite [71]. Nowadays, many types of camphor soap are available on the market. An interesting preparation is a herbal aloe-camphor soap. Its composition includes, among other things, aloe vera gel, camphor, and honey. Such soap can be used for skin prone to irritation and itching, as camphor has antibacterial and antifungal properties [72].

In addition, the properties of this monoterpenoid have been utilized in the therapy of respiratory disorders. Indeed, it is a component of multicomponent preparations, i.e., nasal and inhalation drops (e.g., Makatussin, Optipect, Sedum Balsam, and Algorhin); inhalation liquids (e.g., Cetix and Cetix Plus); syrups (e.g., Optipect and Halin); capsules (e.g., Pinimenthol); lozenges (e.g., Neo-Angin); and lotions, gels and ointments (e.g., Dracodermalin, Herbolen, Mentoklar, Rhino-tussal, Rhino-tussal S, Transpulmin, and Pulmex) [73].

## 5. Camphor Metabolism

Camphor is a highly lipophilic cyclic terpenoid that undergoes rapid diffusion. It can be absorbed through (i) the gastrointestinal tract (within 5 to 90 min after ingestion), (ii) the skin, and (iii) mucous membranes [74,75]. In the body, camphor is metabolized in the liver through oxidation and glucuronidation processes. Subsequently, the inactive metabolites formed as a result are excreted by the kidneys. When applied to healthy skin, its absorption volume is relatively low compared to the rate of the process [34]. Research conducted by Martin et al. revealed that the 8-h application of two patches of Satogesic™ Medicated Adhesive patch from Sato Pharmaceutical Company that contained a 1% solution of camphor led to its release into the bloodstreams of study participants below the limit of detectability. However, administration of 4 or 8 patches, corresponding to 2% and 4% solutions of this monoterpene, respectively, contributed to its penetration through the skin at levels of 26.8 ± 7.2 ng/mL vs. 41.0 ± 5.8 ng/mL (4 patches vs. 8 patches), with a mean half-life of up to 5.6 ± 1.3 h, whereas the standard half-life (3 µg/mL anethole in ethanol) does not exceed 167 min [76]. Therefore, the use of camphor on the skin should be subject to strict regulations. Poisoning from this monoterpene due to contact with the skin is commonly observed in children, especially in Asia, where there are no strict regulations regarding its use [75].

In the case of oral administration of camphor, high concentrations have been detected in the fetal brain, liver, kidneys, and blood, as well as in the amniotic fluid. Symptoms of camphor poisoning include blurred vision, nausea, vomiting, colitis, dizziness, delirium, cardiac muscle spasms, difficulty breathing, seizures, and even death [11]. The lethal dose of camphor in adults ranges from 50 to 500 mg/kg. In contrast, for children, it is 0.5–1 g, and for infants, it is 70 mg/kg [74].

Although the first studies on camphor metabolism in humans were conducted in the 1960s, it has not been fully elucidated to date [77]. Valuable insights in this field are provided by research conducted by Park et al., which indicated that camphor specifically inhibits nicotinic acetylcholine receptors (nAChR), thereby preventing the secretion of catecholamines. This action may be one of the causes of neurotoxicity, as nAChR are known to play a key role in neuromuscular junctions [78]. Moreover, camphor may modulate the activity of hepatic enzymes involved in drug metabolism. Indeed, it has been demonstrated that a drug containing this compound, when applied to the skin for cold relief, caused a transient elevation in serum liver enzymes in a 2-month-old infant [79,80]. Additionally, in the case of Swiss Albino mice treated with camphor at a dose of 300 mg/kg, an increase in hepatic enzyme activity, including CYP (cytochrome P450), cytochrome b5, aryl hydrocarbon hydroxylase, and glutathione S-transferase, was observed, leading to a significant elevation in reduced glutathione levels [34].

Identification of camphor biotransformation products in humans is only possible in cases of acute poisoning. In the urine of patients admitted to the hospital due to intoxication after ingesting 6–10 g of camphor, the main metabolites identified included camphor hydroxylated at positions 3, 5, 8, and 9, as well as further oxidation products such as 5-ketocamphor and carboxylic acids resulting from the oxidation of 8- or 9-hydroxycamphor. Interestingly, the presence of isoborneol in urine was the result of the microbiological reduction of camphor [81]. Within in vitro studies, (-)-camphor was incubated with human liver microsomes in the presence of an NADPH-generating system. Hydroxylation of this compound to 5-*exo*-hydroxycamphor was observed, which is also the main biotransformation product of camphor in dogs, rabbits, and rats [82].

**Figure 2:**
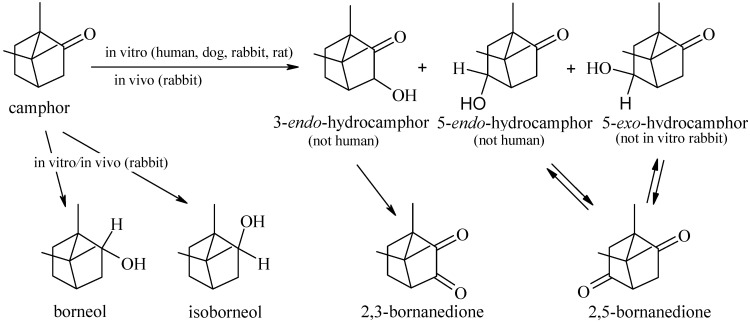
Camphor’s biotransformation pathways in humans and animals.

Numerous studies, extending back more than 150 years, have provided valuable information regarding the transforming ability of camphor in the animal body. Indeed, the presence of the glucuronide derivative of hydroxycamphor in the urine of dogs was demonstrated, as well as 3-hydroxycamphor, 5-hydroxycamphor, and *cis* and *trans*-π-hydroxycamphor, after the administration of (+)-camphor with food [83]. On the other hand, in the case of rabbits, there was additionally a stereospecific *endo*-reduction of camphor to borneol, and a small amount of isoborneol in the liver cytosol [84,85]. The biotransformation abilities that camphor represents in the human and animal organisms are shown in Figure 2.

In addition, in vitro studies showed that insect cells are capable of oxidizing (-)-camphor. The experiment was conducted in a human liver microsome system—an NADPH-producing system—with human recombinant enzymes (CYP1A1, CYP1A2, CYP1B1, CYP2A6, CYP2B6, CYP2C8, CYP2C9, CYP2C19, CYP2D6, CYP2E1, and CYP3A4). It was proven that only CYP2A6 was capable of catalyzing the hydroxylation of camphor [82,86].

Another significant step in camphor metabolism is the conjugation of its oxidation products with glucuronic acid in the liver, followed by excretion through the kidneys. The transferase responsible for conjugating hydroxylated derivatives of camphor is not known. However, it is understood that both borneol and isoborneol undergo extensive glucuronidation via uridine 5′-diphosphoglucuronosyltransferase UGT2B7 to form borneol-2-*O*-glucuronide and isoborneol-2-*O*-glucuronide, respectively. Subsequently, multi-drug resistance-associated proteins MRP3 and MRP4 facilitate the efflux of glucuronides into the bloodstream. They are then primarily eliminated by the kidneys, through both glomerular filtration and tubular secretion via organic anion transporters OAT3 and OAT4 [87].

It is worth noting that camphor can significantly affect the permeation of other drugs through the skin. It has been shown that this terpenoid facilitates the penetration of lipophilic drugs through the stratum corneum of the epidermis, including indomethacin, lidocaine, aspirin, antipyrine, tegafur, and 5-fluorouracil [88,89]. The mechanism of action was elucidated by studies conducted by Cui et al., which demonstrated that (+)-camphor is capable of disrupting the lipid bilayer of the rat skin, thereby increasing the partition coefficient of propranolol (PHCl) into the stratum corneum of the epidermis [90]. This ability of camphor has significant clinical implications, as propranolol, a non-selective β_1_- and β_2_-adrenergic receptor antagonist, is used in the treatment of hypertension, coronary artery disease, and tachyarrhythmias [91].

## 6. Molecular Activity of Camphor

Camphor is known as a natural bioactive compound with wide spectrum of biological activities. Those most frequently described are antibacterial, antifungal, antioxidant, anticancer, analgesic, and anti-inflammatory properties. The activities are usually described for EO in which camphor is a major/dominant compound. Due to its toxicity at higher concentrations, there are rarely reports on the biological activity of pure camphor.

It has been shown that the EO obtained from *Commiphora ornifolia* (Balf. fil.) Gillett was characterized by a high content of oxygenated monoterpenes (56.3%), of which camphor constituted 27.3%. It has shown significant influences on antimicrobial activity against both Gram-positive and Gram-negative bacteria. Unfortunately, investigated EOs demonstrated weak antioxidant abilities, which were calculated based on their capability to reduce 2,2-diphenyl-1-picrylhydrazyl (DPPH) [92]. Yuceturk and co-workers observed an increase in the ability to reduce the DPPH radical with a higher concentration of EO of *Thymus convolutus* Klokov. The EO consisted of 66 compounds, in which camphor was the main component (16.6%, GC/MS analysis). Authors calculated that 33.39 ± 0.25% DPPH was scavenging at a concentration of 1 mg/mL of EO. Furthermore, antiproliferative activity of EO of *T. convolutus* on human liver adenocarcinoma Hep3B cells was observed at concentrations of 250 and 125 µg/mL, although camphor was cytotoxic in high concentrations (1 mg/mL, 500, 250 and 125 µg/mL). The inhibitory effect of EO on the human colorectal adenocarcinoma HT-29 cell line at concentration of 500 µg/mL was determined. Moreover, neither EO nor camphor exhibited a significant effect on the proliferation of human umbilical vein endothelial cells (HUVEC) [93]. The antitumor activity of novel camphor-based pyrimidine derivatives was evaluated in the Zhang et al. study. It has been proven that one of the tested derivatives showed similar activity to etoposide in the inhibition of growth of the following cell lines: MDA-MB-231 (breast cancer), RPMI-8226 (multiple myeloma), and A549 (non-small cell lung cancer NSCLC). At the same time, the compounds showed lower cytotoxicity than the comparator in relation to the tested line of normal human cells (GES-1). Moreover, at the molecular level, an increase in the formation of apoptotic proteins Bax, cytochrome C, and caspase 3 was observed, although the formation of the anti-apoptotic protein Bcl-2 was significantly decreased. The proper anti-oncogenic effect was present in ROS accumulation in the cell and toxicity to mitochondria, which in turn induced the apoptosis pathway [94].

Research performed by Rawat et al. consisted of obtaining EO of *Hedychium spicatum* Sm. from Himachal Pradesh of India, in which the content of camphor was 35.6%. Studies have shown that the EO suppressed 33.57% of inflammation at 100 mg/kg b.wt. dose level, which is comparable to the standard drug ibuprofen (40.06%) [95]. Scientists from Iran tested the analgesic activity of the EO from *Artemisia sieberi* Besser, which possess 31.2% of camphor. In studies, 1 mg/kg dose of the EO significantly reduced carrageenan-induced paw edema in rats by 74.3%, which is very close to the results for the standard drug diclofenac sodium (50 mg/kg) [96]. Li and co-workers also studied the analgesic effect of camphor. Based on neuropathic pain models in mice, the scientists concluded that camphor can alleviate symptoms of hyperalgesia [6]. The molecular anti-inflammatory mechanism of EO from *Blumea balsamifera* (camphor ~9%) was also evaluated. The EO was used as a suppressor in an in vitro model of LPS (lipopolysaccharide)-related activation of macrophages of the RAW 264.7 line. The decrease in the synthesis of proinflammatory cytokines (TNF-α, IL-1β, and IL-6) and the weakening of the medio-signaling pathway mediated by NF-κB (nuclear factor kappa B) and TLR-4 (Toll-like receptor 4) were revealed. Attenuation of signaling pathways led to, among other effects, inhibition of the NLRP3 inflammasome. [97].

Due to the ability of camphor to induce the proliferation of human primary dermal fibroblasts, which are responsible for the expression of collagen IA, collagen IIIA, collagen IVA, and elastin, preparations containing camphor can be used in cosmetology as anti-wrinkle agents [98].

### Molecular Effects of Camphor-Containing Essential Oils on Pathogen Cells

The Pejčić et al. study evaluated the effects of *S. officinalis* (16.6% camphor) and *Ocimum basilicum* EOs on *P. aeruginosa* biofilm-forming capacity, motility, and pyrocyanin production. MIC values of camphor-containing oil oscillated at 5–20 mg/mL against all tested isolates (including skin-wound-infection isolates). The ability of the EOs to inhibit biofilm formation was determined to be 84.1–99.6% in the concentration range of 5, 10, and 20 mg/mL. The ability to inhibit the growth of previously formed biofilms was also assessed as follows: 20 mg/mL (12.4–95.7% inhibition), 10 mg/mL (0.4–94.5% inhibition), and 5 mg/mL (11.3–94.2% inhibition). A decrease in flagellum-dependent forms of movement (swimming) of bacteria was also observed by 97% (MIC) and 81% (1/2 MIC), respectively. Similar results were observed for other forms of movement (twitching and swarming). Sage oil inhibited the production of pyrocyanin by more than 58.8% [99]. Bajalan et al. evaluated the antibacterial activity of EOs obtained from *Rosmarinus officinalis*. At the molecular level, an increase in the permeability of cell membranes of pathogens and a loss of metabolically important ions was observed [100]. In the phototoxicity test of EOs, including *Lavandula stoechas* (36.69% of camphor; white light 470, Lumen/40 watt), the ZOI increased in the case of *K. pneumoniae* (ZOI in the dark = 2.2 mm, ZOI after irradiation = 4.07 mm) [101].

Due to numerous reports on the antibacterial properties of camphor-rich essential oils against gram-negative bacteria, we have presented a summary (assessed by microdilution method (MIC) and Kirby-Bauer method) in Table 2 and Table 3.

The activity of the EO from *Artemisia judaica* (16.1% camphor) against pathogenic fungi was assessed in addition to its activities against *Candida albicans* ATCC 10231, *C. parapsilosis* ATCC 90018, and *C. tropicalis* ATCC 13803. The oil showed a significant inhibitory effect on germ tube formation in *C. albicans* with 80% filamentation inhibition at 0.16 μL/mL concentration. In similar tests, *C. albicans*, *C. tropicalis*, and *C. krusei* obtained MIC and MFC values that were both at the level of 1.25 μL/mL, whereas *C. parapsilosis* obtained MIC = 1.25 μL/mL and MFC = 2.5 μL/mL. At a concentration of 1/8 MIC (0.16 μL/mL), there was a decrease in filamentation of about 80%. Similarly, a significant decrease in germ tube formation was observed at the already mentioned concentration (0.16 μL/mL). A decrease in biofilm-biomass formation by more than 50% was also observed at an oil concentration of 2.5 μL/mL [126]. Cedar leaf-derived EO, as well as camphor, which is its main component, significantly inhibited the formation of *C. albicans* DAY185 *hyphae*, thus reducing the formation of biofilm. Transcriptomic analyses showed that camphor and fenchyl alcohol reduced expression levels of *ECE1*, *ECE*2, *RBT*1, and *EED*1 genes specific for *hyphae* and biofilm-associated functions. In addition, camphor and fenchyl alcohol reduced the virulence of *C. albicans* in the *Caenorhabditis elegans* nematode-infection model [127]. The results of Ivanov et al.’s study showed the potential of camphor to reduce the virulence of *C. albicans*, i.e., biofilm formation and *hyphae* formation. Camphor additionally regulated the level of Cdr1- and Cdr2-efflux pumps, which belong to ATP-binding cassette transporters and play roles in the development of resistance to azole drugs. It did not affect the expression level of the *ERG1* gene, which is involved in the biosynthesis of ergosterol. The beneficial antifungal activity of camphor was achieved at an amount that was non-toxic to porcine liver cells, making it a promising antifungal compound for future studies [128].

The antifungal activity of camphor-containing oils has also been well documented. A summary of the activities of oils against *C. albicans* is presented in the form of Table 4 and Table 5.

## 7. Mechanisms of Camphor Action on the Skin

Scientists are looking for the therapeutic effect of camphor on human skin. It is used, among others, in sunscreen creams as 4-methylbenzylidene camphor [145].

Camphor affects fibroblast proliferation by inducing phosphorylation of the PI3K/AKT and ER pathways. It prolongs the lifespan of fibroblasts in a dose-dependent manner at concentrations of 32.5, 65, 130, and 260 μM, which is responsible for the production of free radicals. The activity of elastase decreases and, consequently, the amount of elastin increases, also in a dose- and time-dependent manner. Its action inhibits skin aging by acting on beta-galactosidase, which is a marker of this process because it accumulates in the lysosomes of aging cells. The cleavage of X-Gal into blue dye was investigated with 600 and 800 μM H_2_O_2_, which led to an increase in the number of SA-β-gal-positive cells. At the same time, 600 and 800 μM H_2_O_2_ together with camphor were used in the same trial, which led to a reduction in the number of SA-β-gal-positive cells by 65% and 50%. This indicates protection of skin fibroblasts against aging [98]. Camphor also prevents the thickening of the epidermis and subcutaneous fat tissue [146].

Camphor affects several types of receptors: heat-sensitive TRPV1, cold-sensitive TRP-M8, and heat-sensitive TRPV3, and it inhibits TRPA1. TRP ion channels contain segments that are thermosensitive, called thermoTRP. Its impact on TRPV1 is linked to a desensitizing effect, resulting in analgesia. Activation of TRPV3 occurs at a temperature of 39 °C, and these receptors are found in keratinocytes, the brain, and the spinal cord [147,148].

**Figure 3:**
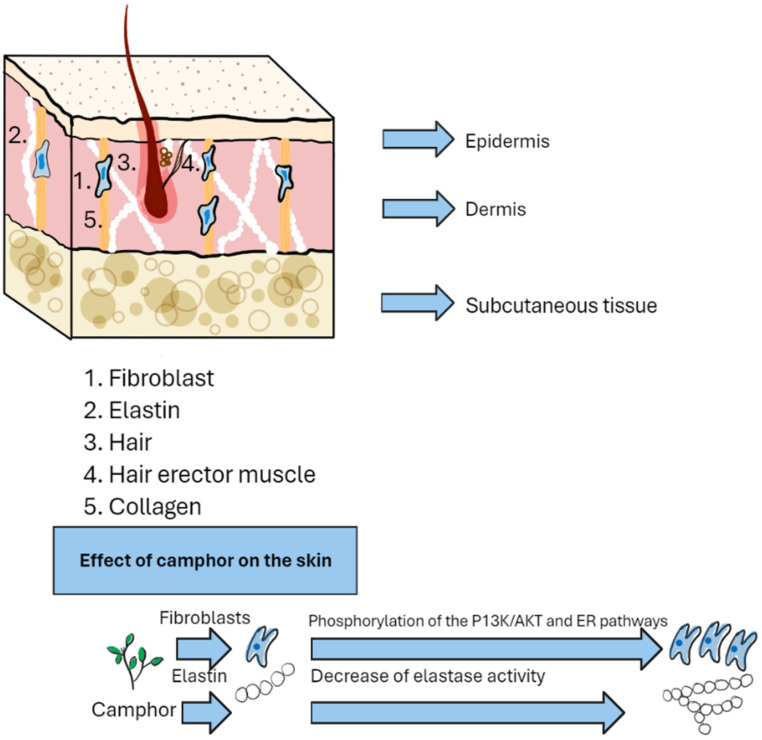
Simplified skin model and camphor effect.

The impact of this compound on blood circulation and temperature perception, including heat and cold, has also been investigated. Camphor increases the sensory experience when the skin temperature is between 33 to 43 °C or 33 to 18 °C. The feeling of heat or cold is perceived more strongly by people after using this compound [149]. An experiment was used in which vaseline containing 5%, 10%, and 20% camphor was placed on the skin of the subjects’ forearms. It was shown that 5% camphor (with vaseline) caused a feeling of cold in the subjects with a delay of about 2 min and that lasted about 5 min, unlike vaseline itself (camphor 0%), which did not cause a feeling of cold. Higher concentrations (10% and 20%) also elicited a cold sensation lasting 9 and 7 min, respectively. Subsequently, subjects reported a sensation of warmth with delays of 3, 7, and 13 min for 5%, 10% and 20% concentrations, respectively, after the cessation of the cold sensation. Blood flow in the skin and muscles also increased with a time delay [150]. It also caused a burning sensation [151]. Camphor is a TRPV3 activator and it has been tested in the epidermal tape peel test. At temperatures of 34 and 42 °C, it did not show faster regeneration of the damaged layer. The temperature range from 36–40 °C showed accelerated regeneration in each case [152]. Applying camphor to the skin is associated with its excretion in urine [153]. Table 6 shows the molecular actions of camphor on the skin and their effects. Furthermore, Figure 3 shows a schematic mechanism of action of this compound specifically in the context of the skin.

### Methods of Treating Skin Diseases

EO containing *Cinnamomum camphora* are used as a natural remedy for soothing skin inflammation [41].

The effect of camphor on penetration into the epidermis was examined. This is important considering the possibility of better absorption of drugs when applied to the skin at the same time. It has been proven to have a weak effect on transepidermal water loss, which is a positive phenomenon. Camphor increases penetration through the skin layers [74,89]. For example, 5% camphor has been shown to improve the transdermal penetration of carvedilol in studies performed on pig skin [155]. Studies were also carried out on rats, which proved better absorption of propanolol hydrochloride when using camphor [90]. In vitro and in vivo studies (on rabbit skin) showed increased absorption of ondansetron when administered with camphor in the form of a gel. It is an antiemetic drug used, among other uses, in chemotherapy [156].

Positive uses of camphor on the skin have an impact on the treatment of atopic dermatitis (AD). The anti-inflammatory effect of this compound has been demonstrated through the phosphorylation of transcription activator 1, Janus kinase signal transducer, and extracellular signal-regulated kinase 1/2. The synthesis of chemokines, which play a significant role in the pathogenesis of AD, was reduced [4].

Combinations of camphor and other natural compounds are also used to treat skin diseases. The use of sesame oil and honey in the treatment of second-degree burn wounds in rats is interesting. The described combination accelerated the healing process [157]. The use of the synergism of camphor with *Lavandula latifolia* was used in a checkerboard study against *S. aureus* and *L. monocytogenes*, showing better inhibitory effects against these microorganisms [55].

The combination of camphor and menthol has been used with positive effect in the treatment of itching, taking advantage of the ability of camphor to stimulate the TRPV3 receptor [158]. Camphor oil with or without glycerol dilution worked very well on facial demodeciodosis and scabies, giving complete cures in concentrations of 100%, 75% and 50% [159].

The effect of phenocamphor on mycosis of the feet, legs, and armpits caused by dermatophytes was also examined. A satisfactory effect was achieved, with a low recurrence rate [160].

Camphor, being a component of UV filters, contributes to protection against harmful radiation. To prevent the degradation of this compound (4-MBC) after dermal administration, this compound was complexed with methyl-β-cyclodextrin (RM-β-CD), which improved its effectiveness [161]. However, a case was described of a 71-year-old man who used cosmetics with a UV filter and was diagnosed with photocontact allergy to 4-methylbenzylidene camphor. The patient had been using these agents for several years [162].

An interesting combination finding is that a cream containing camphor, chondroitin sulfate, and glucosamine sulfate was used for knee osteoarthritis with better results than the placebo group. Improvement was observed after treatment with the preparation after just 4 weeks [163].

To sum up, camphor has numerous applications related to healing effects on skin diseases, and it improves the penetration of drugs into the epidermis.

However, there are reports of a negative impact on the endocrine system, as well as on the kidneys, lungs, testicles, and liver, so new solutions are being sought that will limit the penetration of the compound itself into the body. The doses used in sunscreens do not cause significant hormonal changes. Interestingly, one of the solutions being investigated is enclosing 4-MBC in microspheres [153,164,165]. The lethal dose for camphor is about 3.5 g, although the toxic dose is about 2 g [166]. However, even large amounts applied to the skin are usually not high enough to cause poisoning. One case of chronic cutaneous administration of camphor was observed, resulting in granulomatous hepatitis [10]. There are also two cases described in a hospital in the Bronx, New York, of the administration of large doses of this compound orally and one of administration to the skin, which caused epileptic seizures in children [167]. Camphor may cause dermatological side effects when applied to the skin. These include pruriginous eruptions, erythematous, and papulous oedematous [168].

## 8. Antimicrobial Activity of Camphor

In an era of increasing resistance of microorganisms to the antibiotics used in therapy, an important feature of the natural compounds supporting treatment is their antimicrobial activity. These properties of camphor have been confirmed against both aerobic and anaerobic bacteria, as well as against fungi. In this review, we collected information on microorganisms (bacteria and fungi) that are potential etiological agents of skin infection. We mainly selected those that are relatively easy to transmit in either the outdoor or hospital environment.

### 8.1. Interactions of Camphor-Containing Essential Oils with Antimicrobial Drugs

Potential pharmacological interactions between camphor and *Lavandula latifolia* EO were evaluated against *S. aureus* ATCC 25923. Based on the microdilution method, the MIC of the combination was determined at 0.16–20.0 mg/mL. The high activity of the combination of EOs and (+)-camphor (concentrations 0.31 + 1.25 mg/mL, respectively) was determined to be synergistic [55]. The EO of *Artemisia herba-alba* (32% camphor) was highly active against the MRSA S19 strain, obtaining a MIC value of 1.2 μL/mL. Moreover, synergistic properties of the combination of EOs with cefoxitin against staph were indicated [24]. The synergistic interaction was also detected in the case of *Croton tetradenius* EO with meropenem and ciprofloxacin against *S. aureus* [116]. Grimsey et al. examined the antibacterial activity of *Combretum* spp. leaf extracts (using the Kirby-Bauer method and microdilutions) against among other MRSAs. The isolate was non-susceptible to all tested β-lactams and was also characterized by a high level of resistance to GEN. It also showed only intermediate susceptibility to ciprofloxacin and chloramphenicol. *C. hereroense* extract showed, in turn, excellent activity against *S. aureus* [124]. The Cutillas et al. study evaluated the antibacterial activity of several *Salvia officinalis* EOs (camphor content in the range 10.7–19.8%) against Gram-positive bacteria. *S. aureus* was found to be susceptible to all tested oils (MIC and MBC—minimal bactericidal concentration—were in the range of 0.6–1.3 μL/mL for oils and >15 mM for camphor alone) [169]. Antibacterial and antibiofilm activities of *Salvia officinalis* and *Origanum vulgare* EOs against *S. pyogenes* ATCC 19615 and 49399 was also evaluated. Sage EO contained camphor at concentration of 16.6%. Promising results were obtained, and the MIC of the EOs was 0.5 mg/mL and was identical to the value of MBC against planktonic forms of bacteria. In addition, the oil showed at the same concentration-dependent inhibition seen for the formation of *S. pyogenes* biofilm for both strains (0.25 mg/mL) as well as leading to the eradication of an already produced pathogen biofilm (MBIC [minimum-biofilm inhibitory concentration] was established at 0.5 mg/mL) [170]. The beneficial interaction profile of essential oils rich in camphor is associated with their antibacterial properties against Gram-positive bacteria. The activity of the described substances (microdilution method and Kirby-Bauer assay) is summarized in the form of tables (Table 7 and Table 8).

Interesting reports on the activity of the *Artemisia herba-alba* EO (32% camphor) in relation to imipenem-resistant *A. baumannii* S3310 (IRAB) include the Bekka-Hadji et al. study. Moreover, the synergistic properties of the combination of oil with ticarcillin-clavulanate, cefotaxime, imipenem, and nalidixic acid against IRAB have been demonstrated. This is an important premise in the era of increasing antibiotic resistance [24]. Another article describes the antimicrobial activity of *Salvia officinalis* EO in relation to various pathogenic bacteria, including *A. baumannii*. Camphor was the predominant component of the EO, obtaining a concentration value of 18.72%. Interestingly, the oil showed a synergistic effect in combination with cefazolin in relation to several pathogens (*S. aureus*, *K. pneumoniae*, and *A. baumannii*) [176]. In addition, the synergistic nature of the interaction of the *C. tetradenius* EO with meropenem and ciprofloxacin for *K. pneumoniae* was detected [116]. Encouraging results were also obtained in the field of antibacterial activity for that of *R. officinalis* EO against fluoroquinolone-resistant *E. coli* (*gyrA* and *parC* mutations) [122]. In the Asili et al. study, the antibacterial activity of *Artemisia annua* EO (content of ~30% camphor) was assessed in relation to MDR *E. coli* ESβL. MIC values of *A. annua* EO in relation to five clinical isolates of *E. coli* ESβL were at the level of 2.5–10 mg/mL [115]. Antibacterial activity of *C. tetradenius* EO (camphor content 13.95%) in relation to *E. coli* ATCC 25922 was described in the Siqueira et al. study. Importantly, the synergism of the EO with meropenem in the case of *E. coli* was demonstrated [116].

Due to the fact that drug resistance is a significant problem in the effectiveness and speed of treatment of skin infections, in Table 9 we summarize information on the resistance of the bacterial and fungal strains we analyzed, including the mechanism of resistance.

### 8.2. Antimicrobial Activity of Novel Camphor-Based Derivates

The activity of silver complexes with camphor–imine derivatives was also assessed against the *E. coli* ATCC 25922 strain. One of the compounds was characterized by strong activity against a pathogen, with an MIC value of 7.2 ± 0.1 µg/mL. The study tested derivatives also against the *S. aureus* strain Newman. One of the imine derivatives showed an excellent activity profile against *S. aureus* (MIC = 9.3 ± 1.1 μg/mL). The study also proved higher antibacterial activity on the part of camphor sulfonimine derivatives than imines. The derivatives mentioned were also tested in relation to *P. aeruginosa* 477. The MIC for this pathogen obtained by one of the imine derivatives was 3.4 ± 0.1 μg/mL [177]. Novel camphor-based organic derivates from the Peraman et al. study showed that camphor and camphorosulfonic acid derivatives are characterized by a wide spectrum of antibacterial activities, including against MRSA. The presence of the camphor group resulted in an excellent antibacterial activity of the preparation (MIC for MRSA at the level of 24 μM). Camphor quinoxalin-2,3(1H,4H)-dione (MIC = 24 μM) and isatin-based derivates obtained a MIC values of 24 μM, 51 μM, and 82 μM, respectively, against MRSA and *P. aeruginosa* ATCC 27853. In the study, camphor-linked biphenyl quinoxalin-6-sulfonamide obtained promising results against *P. aeruginosa*, *A. baumannii*, and MDR *K. pneumoniae* ATCC 700603 (ESβL- extended spectrum β-lactamase isolate), reaching a MIC value of 16 μg/mL. Camphor and camphor-sulfonic acid derivatives were also highly active against an MDR *C. albicans* ATCC 90028 isolate. The best properties in relation to yeasts were shown by benzoin and salicylic derivatives of camphor obtaining MIC at the levels of 16 μg/mL and 8 μg/mL, respectively (positive control amphotericin B MIC = 2 μg/mL) [178].

Sancineto et al. revealed the antimicrobial activity of organoselenic compounds (including camphor diselenide) against biofilms and planktonic forms of *S. aureus* ATCC 29213 and *S. pyogenes* ATCC 20565 as important etiological factors of skin wound infections. Camphor diselenide showed moderate activity against *S. pyogenes*: MIC = 31.25 mg/L (positive control gentamycin, MIC = 2.19 mg/L). Camphor diselenide also showed an excellent biofilm inhibitory profile against *S. pyogenes* (including subinhibitory concentrations). The antimicrobial activity of camphor diselenide against biofilm and planktonic forms of *S. aureus* ATCC 29213 was also revealed. The in vitro study showed that MIC for *S. aureus* was at the level of 250 mg/L. This derivative was characterized by a strong biofilm inhibitory profile even in subinhibitory concentrations of 0.5× MIC. The antifungal properties of camphor diselenide were also revealed against *C. albicans* Sc5314. The most preferred MIC profile was that obtained by one of the derivatives (MIC = 31.25 mg/L) compared to the positive control FLC, for which MIC = 0.25 mg/L. One of the compounds at a concentration of 50 mg/L showed a decrease in biofilm formation by about 60% [27]. The Zhang et al.’s study evaluated the antibacterial properties of newly synthesized camphorylpyrimidine-amine derivatives containing bicyclic monoterpene groups. One of the derivatives (*N*-(2,4-Difluorobenzyl)-4-(4-methoxyphenyl) -8,9,9-trimethyl-5,6,7,8-tetrahydro -5,8-Methylquinazolin-2-amine) of camphor showed an interesting activity profile in relation to the tested bacteria *K. pneumoniae* (MIC = 32 μg/mL, equal to positive control of ACN). Optimism is inspired by the excellent activity of the derivative against *K. pneumoniae* and *P. aeruginosa*, which was comparable or higher than that of aminoglycosides. The preparation showed an MIC for *E. coli* at 8 μg/mL vs. 2 μg/mL for the positive control can, and 16 µg/mL for *P. aeruginosa versus* 32 µg/mL for ACN. Zhang et al. also described significant activity of one of the compounds against MRSA isolates (MIC = 8 μg/mL vs. positive control CAN’s MIC = 1 μg/mL) and against pathogenic fungi *C. albicans* (MIC = 32 μg/mL vs. ketoconazole’s MIC = 16 μg/mL) [94].

Carvalho et al. showed an interesting profile of antibacterial properties of silver complexes with imine and sulfoimine derivatives of camphor in relation to, among other microbes, *P. aeruginosa* 477. One of the camphor–sulphonyl–imine complexes obtained the best anti-pseudomonal properties (MIC = 36 μg/mL), which makes it an interesting option for further research in the era of increasing antibiotic resistance among *Pseudomonas aeruginosa* strains [123]. A similar report comes from the Cardoso et al. study, in which researchers assessed the effects of camphor–imine complexes with silver ions (Ag^+^) against microbes such as. *P. aeruginosa* and obtained the encouraging MIC result of 19 μg/mL for one of the derivatives [179]. Another study showed the promising properties of the camphor-based 2,4-disubstituted 1,3-thiazoles in the terms of their activity against *C. albicans*. The MIC for yeasts was defined in the range of 0.12–0.98 μg/mL, which makes their activity comparable to that of tested fluconazole [180].

## 9. Future Perspectives of Antimicrobial Usage of Camphor Derivates

Another study determined camphor activity (concentrations 30–50%) in combination with metronidazole [Metronidazole-Loaded Camphor-Based In Situ Forming Matrix] for consideration as a therapeutic option against bacteria involved in periodontitis. The combination of 40% camphor with 5% triacetin has been shown to prolong the release of metronidazole from the substrate for up to 6 days. Activity against *S. aureus* ATCC 6538 was determined by disc diffusion assay. The test with the best parameters showed a ZOI value for *S. aureus* equal to 15.03 ± 0.6 mm, (control sample ZOI was ~23.3 mm) [181]. The Rani et al. study evaluated the antimicrobial activity of a camphor-enriched food film based on soy protein. No significant antimicrobial activity (against *E. coli*, *Listeria monocytogenes*) was demonstrated for the film as opposed to that of a 1% camphor solution alone. It has been shown that after incubation (12–16 h) of film fragments in agar, there was no significant decrease in optical density. In turn, in the case of a 1% camphor solution, this density was observed to decrease by 12.5% (*L. monocytogenes*) and 62.5% (*E. coli*). Although the study does not prove that the innovative food film had antibacterial properties, it does validate earlier reports of significant camphor activity against *E. coli* [182]. Interesting findings came from an article by Santos et al. on the use of a coating made of diamond-like carbon foil with a built-in camphor molecule. The effectiveness of a coating applied to the surface of polyurethane was assessed to combat *C. albicans’* biofilm (including yeast colonization of vascular catheters). ATCC 10231 was the strain used in the study. A significant reduction in the formation of biofilm by the fungus at the level of 91–99% has been shown, which makes the results very promising [183].

**Figure 4:**
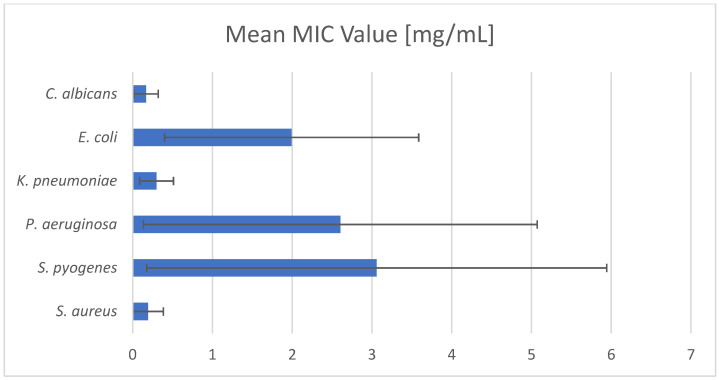
Camphor’s averaged antimicrobial and antifungal activities (based on unified data from the literature collected from articles described and cited in Table 4, Table 5, Table 6, Table 7 and Table 8).

Figure 4 summarizes the average MIC values from the literature data analyzed in this review. Only those strains for which at least three antimicrobial activity values were found were considered. The very high standard deviation (SD) of the compared results is due to the fact that the analysis included studies focusing on different plants that include camphor. Nevertheless, based on the graph, it becomes clear that camphor shows an antimicrobial tendency against both Gram-positive and Gram-negative bacteria, as well as against the fungus species *C. albicans*. Therefore, its activity covers the vast majority of the panel of pathogens responsible for both primary and secondary skin infections. Thus, the preventive effect of camphor in skin disorders is very promising, not only as an additive to cosmetics, i.e., lotions, shampoos, but especially in medicine as (i) an antiseptic to be applied directly to the skin and (ii) a disinfectant applied to objects in direct contact with the skin.

In addition, camphor, as an additive in small amounts to cosmetic products applied directly to the skin, boasts popularity. It has already found use in foot care cosmetics, such as Gehwol warming lotion and FcSynergy ointment. Its beneficial effects are also utilized in the production of body care cosmetics, such as Flos-lek gel, Ilcsi cleansing concentrate, and Gehwol herbal salt. Camphor is often an ingredient in anti-acne products, e.g., Tołpa. It is also added to shampoos, e.g., Trico Botanica, as well as antiperspirants and perfumes. Because of its wide-ranging effects (i.e., relaxing, warming, relaxing, pore-opening, pH-restoring, and purifying), camphor is a compound with many uses and it surrounds us on all sides.

## 10. Conclusions

In this review article, we proved that camphor is a forward-looking natural compound in medicine. It can be used with great success in the prevention of skin infections. Indeed, it shows activity against both pathogens involved in primary and secondary dermatological infections. Its addition to medical devices applied directly to the skin protects the skin from attack by microorganisms, including those showing multi-drug resistance. In addition, as an ingredient in cosmetic products, it enhances their antiseptic effect and aids in eliminating purulent eruptions. However, due to the toxicity of this compound, future research directions should focus primarily on the aspect of reducing this negative effect. These should include studies searching for associations of camphor with other compounds that would neutralize the toxic effect of camphor while simultaneously enhancing its positive activities, i.e., analgesic, antipruritic, and warming. Moreover, prospective studies around slowing the absorption and/or release of this compound are also very promising. Research supporting the possibility of using camphor in nanotechnology also deserves great attention. The use of nanoparticles carrying camphor are highly promising due to their antitoxic effects. In the future, it is worth focusing on the use of various surfaces (e.g., bandages, sponges) impregnated with nanoparticles filled with camphor. However, this idea still needs many multicenter and multidirectional experiments. There is a need for in vivo studies that would give us the green light regarding the safety of future therapies. Such research could provide guidance on using these nanoparticles not only for preventing skin disorders but mainly as a therapeutic agent in cases of skin barrier disruption. In addition, future research should be based primarily on finding new ways for this compound to interact with, for example, antibiotics on bacterial and fungal cells involved in skin diseases. Therefore, further research using camphor as a wound-healing aid are important. The results of such experiments are even desirable from the point of view of eradicating resistant strains colonizing wounds.

## Figures and Tables

**Table 1:** Plant resources of camphor.

Plant Cultivar	Source of EO	Percentage [%]	References
*Achillea grandifolia*	Inflorescences	10.0–70.5	[46]
	Leaves	5.5–83.2	
*Achillea millefolium* L.	Inflorescences	13.0	[47]
*Artemisia annua*	Leaves and stems	11.4	[48]
*Artemisia haussknechtii*	Aerial parts	11.8	[49]
*Artemisia khorassanica*	Aerial parts	74.2	[49]
*Artemisia sieberi*	Aerial parts	33.6	[50]
*Artemisia spicigera*	Whole plant	29.6	[51]
*Chrysanthemum japonense*	Flowers	47.6	[52]
	Leaves	39.1	
*Cinnamomum camphora*	Leaves	93.1	[33]
	Branch	53.6	
	Wood	53.2	
*Cinnamomum camphora*	Barks	51.3	[53]
	Leaves	40.5	
	Fruits	28.1	
*Lavandula angustifolia*	Leaves and inflorescences	10.7–18.8	[54]
*Lavandula latifolia*	Whole plant	12.2	[55]
*Ocimum basilicum* L.	Aerial Parts	42.1	[56]
*Ocimum kilimandscharicum* Guerke	Leaves	45.9	[57]
*Santolina chamaecyparissus* L.	Aerial part	17.7	[58]
*Salvia fruticosa*	Aerial part	20.3	[59]
*Salvia jordanii*	Aerial part	33.4	[60]
*Salvia officinalis* L.	Whole plant	26.6 northern Albania	[61]
		43.8 southern Albania	
*Salvia rosmarinus*	Aerial part	3.3–42.2	[60]
*Stachys germanica* L.	Aerial parts	52.9	[62]
*Tanacetum parthenium* L.	Aerial parts	53.4–52.9	[63]
	Stems and leaves	47.9–49.6	
	Inflorescence	11.6–11.5	
	Unripe seeds	12.3–12.4	
	Ripe seeds	12.3–10.3	
*Thymus algeriensis*	Whole plant	6.8–19.9 (vegetative stage)	[64]
		8.1–15.7 (flowering stage)	

**Table 2:** Antibacterial activities of camphor-containing essential oils against Gram-negative bacteria (minimal dilution assay).

Bacteria	Plant Cultivar	Camphor Concentration [%]	MIC Value	Ref.
***P. aeruginosa***	*Salvia officinalis*	16.4%	0.125 mg/mL	[102]
	*Salvia officinalis*	16.6%	5 mg/mL	[99]
	*Curcuma aeruginosa*	29.39%	0.125 mg/mL	[103]
			1 mg/mL	
	*Thymus hirtus* ssp. *algeriensis*	19.2%	0.022 mg/mL	[104]
	*Salvia pachystachys*	31.0%	5 mg/mL	[105]
	*Ferula communis*	18.3%	0.156 mg/mL	[106]
	*Rosmarinus officinalis*	18.74%	2 mg/mL	[107]
	*Pulicaria undulata*	44.48%	6.25 mg/mL	[108]
	*Rosmarinus officinalis*	26.5%	2.56 mg/mL	[109]
	*Oncosiphon suffruticosum*	31%	6.4 mg/mL	[110]
***A. baumannii***	*Artemisia herba-alba* Asso	50.7%	10 mg/mL	[111]
			20 mg/mL	
	*Rosmarinus officinalis*	23.04%	12.5 mg/mL	[112]
***K. pneumoniae***	*Lavandula stoechas* ‘avonview’	35.30%	0.39 mg/mL	[113]
	*Lavandula stoechas*	36.69%	0.5 mg/mL	[101]
	*Withania frutescens*	~24–25%	0.006125 mg/mL	[114]
***E. coli***	*Rosmarinus officinalis*	18.743%	1 mg/mL	[107]
	*Thymus hirtus* ssp. *algeriensis*	19.2%	1.8 mg/mL	[104]
	*Rosmarinus officinalis*	26.5%	1.28 mg/mL	[109]
	*Artemisia annua*	~30%	2.5 mg/mL	[115]
	*Croton tetradenius*	~14%	5.6 mg/mL	[116]
	*Withania frutescens*	~24–25%	0.006125 mg/mL	[114]
	*Lavandula stoechas*	36.69%	1 mg/mL	[101]
	-	pure camphor	2.75 mg/mL	[117]

**Table 3:** Antibacterial activities of camphor-containing essential oils against Gram-negative bacteria (Kirby-Bauer assay).

Bacteria	Plant Cultivar	Camphor Conc. [%]	ZOI Value for EO [mm]	EO Content per Disc	ZOI Value for Positive Control	Positive Control Content per Disc	Ref.
***P. aeruginosa***	*Curcuma aeruginosa*	29.39%	5.7	10 µL	7.6	10 µg GM	[103]
	*Withania frutescens*	~24–25%	16.11	10 µL	-	20 µg STR, 1.67 mg AMP	[114]
	*Rosmarinus officinalis*	19.75%	7.0	50 µL	ND	10 µg PCN	[118]
	*Artemisia absinthium*	39.1%	12.0				
	*Tetraclinis articulata*	20.1%	20.0	6 µL	20.0	10 µg CPL	[119]
					15.0	30 µg CHL	
					32.0	30 µg GM	
					-	20/10 µg AMC	
	*Pulicaria undulata*	44.48%	24.0	20 µL	17.0	ND GM	[108]
					16.0	ND TET	
	*Artemisia sieberi* Besser	42.9%	10.3	5 µL	ND	ND	[120]
		41.6%	8.6				
		37.1%	10.3				
	-	pure camphor	10.4	ND	ND	ND	
***A. baumannii***	*Artemisia herba-alba* Asso	50.47%	30.6	6 µL	13.6	10 µg STR	[111]
			27.3		10.0		
	*Rosmarinus officinalis*	23.04%	34.0	ND	-	-	[112]
	*Artemisia herba-alba*	32.0%	15.3	10 µL	14.0	ND CIP	[24]
					12.0	ND AKN	
	*Salvia officinalis*	17.1%	13.67	10 µL	ND	20 µg GM	[121]
***K. pneumoniae***	*Tetraclinis articulata*	20.1%	29.0	6 µL	22.0	10 µg CPL	[119]
					30.0	30 µg CHL	
					39.0	30 µg GM	
					30.0	20/10 µg AMC	
	*Rosmarinus officinalis*	24.82%	13.12	20 µL	6.0	ND OTC	[100]
		21.41%	9.06				
	*Rosmarinus officinalis*	16.99%	8.67	5 mg	28.0	5 mg NAL	[122]
		20.4%	9.0				
	*Lavandula stoechas* ‘avonview’	42.47%	10.0	30 µL	9.30	ND CHL	[123]
		35.30%	8.03				
	*Combretum hereroense*	ND	18.2	10 µL	18.8	30 µg CFX	[124]
***E. coli***	*Rosmarinus officinalis*	16.99%	12.67	5 mg	30.0	5 mg NAL	[122]
		20.4%	13.67				
	*Thymus hirtus* ssp. *algeriensis*	19.2%	30.0	10 µL	15.0	ND STR	[104]
					27.0	ND CHL	
	*Salvia officinalis*	17.1%	16.67	10 µL	ND	20 µg GM	[121]
	*Pulicaria undulata*	44.48%	17.0	20 µL	22.0	ND GM	[108]
					16.0	ND TET	
	*Artemisia sieberi* Besser	42.9%	12.8	5 µL	ND	ND	[120]
		41.6%	12.4				
		37.1%	12.3				
	-	pure camphor	12.91	ND	ND	ND	
	*Thymus algeriensis*	17.45–32.56%	13.0	40 µL	27.0	25 µg AMX	[125]
	*Rosmarinus officinalis*	26.5%	14.2	ND	21.5	ND CIP	[109]
	*Combretum hereroense*	ND	15.8	10 µL	23.2	30 µg CFX	[124]

AKN—amikacin; AMC—amoxicillin/clavulanate; AMP—ampicillin; AMX—amoxicillin; CFX—cefoxitin; CHL—chloramphenicol; CIP—cyprofloxacine; CPL—colistin; GM—gentamycin; NAL—nalidixic acid; OTC—oxytetracycline; PCN—penicillin; STR—streptomycin; TET—tetracycline; ND—not defined.

**Table 4:** Antifungal activities of camphor-containing essential oils against *Candida albicans* (minimal dilution assay).

Plant Cultivar	Camphor Concentration [%]	MIC Value	Ref.
*Salvia lavandulifolia*	29.1%	0.156 mg/mL	[129]
		0.312 mg/mL	
*Curcuma aeruginosa*	29.39%	0.25 mg/mL	[103]
-	pure camphor	0.125 mg/mL	[128]
		0.35 mg/mL	
*Withania frutescens*	24.26%	0.0004 mg/mL	[114]
*Rosmarinus officinalis*	26.5%	0.16 mg/mL	[109]
*Cassia fistula*	~14%	0.313 mg/mL	[130]
-	pure camphor	0.156 mg/mL	
*Tetraclinis articulata*	20.1%	0.268 mg/mL	[119]
*Brocchia cinerea*	~14%	0.0168 mg/mL	[131]
*Plectranthus cylindraceus* Hochst. et Benth	40.93%	0.4 mg/mL	[132]
*Rosmarinus officinalis*	24.3%	0.025 mg/mL	[133]
	15.52%	0.00156 mg/mL	
	18.47%	0.00078 mg/mL	
*Anvillea radiata*	21.41%	0.01031 mg/mL	[134]
		0.02275 mg/mL	
*Artemisia aucheri*	18%	0.4 mg/mL	[135]
*Salvia fruticosa*	21.32%	0.079 mg/mL	[136]
*Achillea grandifolia*	23.4%	0.16042 mg/mL	[137]
		0.525 mg/mL	
*Tetraclinis articulata*	15.97%	0.125 mg/mL	[138]
*Artemisia herba-alba* var. *huguetii*	18.38%	0.00183 mg/mL	[139]

**Table 5:** Antifungal activities of camphor-containing essential oils against *Candida albicans* (Kirby-Bauer assay).

Plant Cultivar	Camphor Conc. [%]	ZOI Value for EO [mm]	EO Content per Disc	ZOI Value for Positive Control	Positive Control Content per Disc	Ref.
*Curcuma aeruginosa*	29.39%	11.6	10 µL	10.54	10 µg COM	[103]
*Ocimum kilimandscharicum* Guerke	52.0–57.2%	4.33–5.33	8 µL	24.33	ND KTC	[140]
		4.66–6.33				
*Achillea odorata* subsp. *pectinata*	45.01	25.33	12.5 µL	18.66	10 µg AMB	[141]
*Pulicaria undulata*	44.48%	23.0	20 µL	14.0	ND NYT	[108]
*Withania frutescens*	24.26%	47.0	10 µL	21.2	ND FLC	[114]
*Suaeda vermiculata* Forssk	28.74%	19.0	50 µL	16.0	10 µg CLT	[108]
*Rosmarinus officinalis*	33.9–41.2%	11.6–14.6	10 µL	28.0	10 µg MIC	[142]
				18.0	20 µg AMB	
*Artemisia annua*	17.0%	8.5	15–30 µL	ND	ND	[143]
*Artemisia siebieri* Besser	42.9%	16.2	5 µL	-	-	[120]
	41.6%	14.2				
	37.1%	16.0				
-	pure camphor	11.4	ND	-	-	
*Thymus algeriensis*	17.45–32.56%	13.0	80 µL	24.0	25 µg ITC	[125]
*Rosmarinus officinalis*	26.5%	18.2	ND	21.0	ND FLC	[109]
		18.6		16.0		
*Tetraclinis articulata*	20.1%	22.0	6 µL	10.0	100 µg AMB	[125]
				18.0	50 µg ECC	
				19.0	50 µg MIC	
				10.0	50 µg CLT	
				0.0	1 µg 5FC	
*Brocchia cinerea*	~14%	42.33	20 µL	21.0	ND FLC	[131]
*Matricaria chamomilla*	16.42%	21.07	10 µL	28.6	100 I.U. NYT	[144]
*Tetraclinis articulata*	15.97%	15.6	10 µL	~10	20 µg FLC	[138]
				~20	20 µg CLT	
*Artemisia herba-alba* var. *huguetii*	18.38%	39.0	4.7 µg	24.7	ND FLC	[139]

AMB—amphotericin B; CLT—clotrimazole; COM—ciclopirox olamine; ECC—econazole; FLC—fluconazole; ITC—itraconazole; KTC—ketoconazole; MIC—miconazole; NYT—nystatin; 5FC—flucytosine; ND—not defined.

**Table 6:** Molecular effects of camphor on the skin.

Molecular Actions of Camphor	Effect	Ref.
Decrease in elastase activity	Increase in the amount of elastin	[98]
Induction of phosphorylation of PI3K/AKT and ER pathways	Inhibition of fibroblast apoptosis	
Action on β-galactosidase	Inhibition of skin aging	
Activation of TRPV1	Desensitizing and analgesic effect	[147]
Activation of TRPM8	Activation of the channel, enhancement of cold sensitivity, inhibition of the response to menthol	[154]
Activation of TRPV3	Heat activation, suggestive sensitizing role	[148]
Activation and inhibition of TRPA1	Agonist–antagonist action, probable role as a cold sensor in nociceptive neurons	[151]

**Table 7:** Antibacterial activities of camphor-containing essential oils against Gram-positive bacteria (minimal dilution assay).

Bacteria	Plant Cultivar	Camphor Concentration [%]	MIC Value	Ref.
***S. aureus***	*Lavandula stoechas*	8.64%	0.01795 mg/mL	[171]
	*Salvia officinalis*	16.4%	0.125 mg/mL	[102]
	*Ocimum tenuiflorum*	31.52%	0.00225 mg/mL	[103]
	*Lavandula stoechas*	36.69%	0.5 mg/mL	[101]
	*Rosmarinus officinalis*	26.5%	0.32 mg/mL	[109]
***S. pyogenes***	*Satureja montana*	29.1%	0.03 mg/mL	[129]
			0.63 mg/mL	
	*Makhlaseh*	49.0–56.9%	8 mg/mL	[172]
			4 mg/mL	
	*Salvia officinalis*	16.6%	0.5 mg/mL	[170]
	*Perovskia abrotanoides*	21.68%	2.0 mg/mL	[173]
	*Lavandula stoechas × viridis* ‘St. Brelade’s’	7.13%	6.25 mg/mL	[113]
***Clostridium* spp.**	*Artemisia annua*	>60%	~1.6 mg/mL	[174]

**Table 8:** Antimicrobial activities of camphor-containing essential oils against *S. aureus* (Kirby-Bauer assay).

Plant Cultivar	Camphor Conc. [%]	ZOI Value for EO [mm]	EO Content per Disc	ZOI Value for Positive Control [mm]	Positive Control Content per Disc	Ref.
*Ocimum kilimanscharicum* Guerke	54.6%	7.66	8 µL/disc	18.0	ND, TET	[140]
	52.0%	6.66				
	57.2%	5.0				
*Lavandula stoechas*	8.64%	12.0	5.83 mg/disc	22.0 24.0	ND, CIP, GM	[171]
		13.0	23.4 mg/disc			
		18.0	35.1 mg/disc			
*Salvia officinalis*	36.92%	8.0–13.0	20 µL/disc	ND	ND	[172]
*Curcuma aeruginosa*	29.39%	22.0	10 µL/disc	7.28	10 µg GM	[103]
*Artemisia sieberi* Besser	2.8–42.9%	12.3–20.6	5 µL/disc	ND	ND	[120]
-	pure camphor	12.0	ND	ND	ND	
*Thymus algeriensis*	17.45–32.56%	18.0	40 µL/disc	10.0	25 µg AMX	[125]
*Rosmarinus officinalis*	26.5%	25.2	ND	30.0	5 µg CIP	[109]
*Cinnamomum camphora*	3%	7.0	ND	15.0	10 µg STR	[175]
	6%	11.0				
	9%	17.0				
*Combretum hereroense*	ND	21.4	10 µL/disc	7.2	5 µg/MET	[124]
				>15.0	10 µg CHL, GM 5 µg CIP	

AMX—amoxicillin; CHL—chloramphenicol; CIP—ciprofloxacine; GM—gentamycin; MET—methicillin; STR—streptomycin; TET—tetracycline; ND—not defined.

**Table 9:** Antibacterial and antifungal activity of camphor-enriched EOs and derivates, including resistance of the tested strains.

Camphor Source	MIC Value	ZOI Value	Pathogen	Resistance Mechanism	Ref.
*Artemisia judaica* EO (16.1% camphor)	1.25 μL/mL	-	*C. albicans*	Resistance to azoles mediated by efflux pumps Cdr1 and Cdr2	[101]
*Artemisia herba-alba* EO (32% camphor)	1.2 μL/mL	-	*S. aureus*	MRSA—methicillin-resistant *S. aureus*, mediated by *mecA* gene resistance to various β-lactam antibiotics	[24]
*Combretum hereroense* EO	0.17 mg/mL				[127]
Camphor quinoxalin-2,3(1H,4H)-dione	24 μM	-			[134]
*Artemisia herba-alba* EO (32% camphor)	1.2 µL/mL	-	*A. baumannii*	IRAB—imipenem-resistant *A. baumannii*	[24]
*Rosmarinus officinalis* EO (~23% camphor)	-	9.33–12.67 mm vs. 30.0 mm nalidixic acid	*E. coli*	Resistance to fluoroquinolones mediated by *gyrA* and *parC* genes mutations	[131]
*Artemisia annua* EO (~30% camphor)	2.5–10 mg/mL	-	*E. coli*	ESβL—extended spectrum β-lactamase	[132]
camphor-linked biphenyl quinoxalin-6-sulfonamide	16 µg/mL	-	*K. pneumoniae*		[134]

## Data Availability

Not applicable.
